# Ultrasound radiomics-based artificial intelligence model to assist in the differential diagnosis of ovarian endometrioma and ovarian dermoid cyst

**DOI:** 10.3389/fmed.2024.1362588

**Published:** 2024-03-08

**Authors:** Lu Liu, Wenjun Cai, Chenyang Zhou, Hongyan Tian, Beibei Wu, Jing Zhang, Guanghui Yue, Yi Hao

**Affiliations:** ^1^Department of Ultrasound Medicine, South China Hospital, Medical School, Shenzhen University, Shenzhen, P. R. China; ^2^Department of Ultrasound, Shenzhen University General Hospital, Medical School, Shenzhen University, Shenzhen, P. R. China; ^3^Department of Information, South China Hospital, Medical School, Shenzhen University, Shenzhen, P. R. China; ^4^National-Regional Key Technology Engineering Laboratory for Medical Ultrasound, Guangdong Key Laboratory for Biomedical Measurements and Ultrasound Imaging, School of Biomedical Engineering, Shenzhen University Medical School, Shenzhen University, Shenzhen, P. R. China

**Keywords:** ultrasound radiomics, artificial intelligence, machine learning, ovarian endometrioma, ovarian dermoid cyst

## Abstract

**Background:**

Accurately differentiating between ovarian endometrioma and ovarian dermoid cyst is of clinical significance. However, the ultrasound appearance of these two diseases is variable, occasionally causing confusion and overlap with each other. This study aimed to develop a diagnostic classification model based on ultrasound radiomics to intelligently distinguish and diagnose the two diseases.

**Methods:**

We collected ovarian ultrasound images from participants diagnosed as patients with ovarian endometrioma or ovarian dermoid cyst. Feature extraction and selection were performed using the Mann-Whitney *U*-test, Spearman correlation analysis, and the least absolute shrinkage and selection operator (LASSO) regression. We then input the final features into the machine learning classifiers for model construction. A nomogram was established by combining the radiomic signature and clinical signature.

**Results:**

A total of 407 participants with 407 lesions were included and categorized into the ovarian endometriomas group (*n* = 200) and the dermoid cyst group (*n* = 207). In the test cohort, Logistic Regression (LR) achieved the highest area under curve (AUC) value (0.981, 95% CI: 0.963−1.000), the highest accuracy (94.8%), and the highest sensitivity (95.5%), while LightGBM achieved the highest specificity (97.1%). A nomogram incorporating both clinical features and radiomic features achieved the highest level of performance (AUC: 0.987, 95% CI: 0.967−1.000, accuracy: 95.1%, sensitivity: 88.0%, specificity: 100.0%, PPV: 100.0%, NPV: 88.0%, precision: 93.6%). No statistical difference in diagnostic performance was observed between the radiomic model and the nomogram (*P* > 0.05). The diagnostic indexes of radiomic model were comparable to that of senior radiologists and superior to that of junior radiologist. The diagnostic performance of junior radiologists significantly improved with the assistance of the model.

**Conclusion:**

This ultrasound radiomics-based model demonstrated superior diagnostic performance compared to those of junior radiologists and comparable diagnostic performance to those of senior radiologists, and it has the potential to enhance the diagnostic performance of junior radiologists.

## 1 Introduction

Ovarian masses encompass a range of pathologies, including both benign and malignant conditions. Sonography, particularly transvaginal sonography, is a primary imaging modality for the initial assessment and differential diagnosis of ovarian masses. Among these masses, ovarian endometrioma and ovarian dermoid cyst are frequently encountered (1, 2). The ultrasound appearance of these two diseases is variable, occasionally causing confusion and overlap with each other (3). Accurately differentiating between these two conditions is of clinical significance, given the distinct clinical management approaches for each.

Ovarian endometrioma occurs when ectopic functional endometrial glands and stroma within the ovary bleed, resulting in the formation of a cyst (4). The characteristic ultrasound features of an endometrioma include homogenous low-level or ground glass internal echoes (5). However, Guerriero et al. (6) reported that in women aged 18−24 years, 11% of endometriomas and in women aged at least 45 years, 21% of endometriomas did not exhibit typical features, with even higher rates in the postmenopausal population. Van Holsbeke et al. (7) reported that nearly 50% of endometriomas displayed ultrasound features other than the typical “unilocular cyst with ground glass echogenicity of the cyst fluid.” Additionally, the ultrasound features of endometriomas can overlap with those of other conditions such as hemorrhagic cyst, dermoid cyst, and cystic ovarian neoplasms, making differentiation challenging (3). Asch and Levine (8) revealed that only 60.3% of endometriomas measuring at least 2 cm in greatest dimension were correctly diagnosed prospectively by sonography. This is attributed to the highly variable ultrasound appearance of endometriomas, which can be influenced by the degradation of blood over time (3).

Ovarian dermoid cyst, also known as ovarian mature cystic teratoma, has a reported incidence ranging from 1.2 to 14.2 per 100,000 women, making it the most common type of ovarian tumor (9). It accounts for 11% of all ovarian tumors and 69% of all germ cell tumors (10). It is composed of mature tissues derived from two or three embryonic layers, including mature endodermal, mesodermal, and ectodermal tissue (11). The typical ultrasound features of dermoid cysts described in the literature are the presence of “dots and/or lines” and the “echogenic white ball” (12, 13). However, in a study by Heremans et al. (14), one or both of these typical features was present in 81.1% of dermoid cyst cases, with some cases showing none of the typical ultrasound features. The study involved 454 patients with pathologically confirmed dermoid cysts who underwent transvaginal sonography performed by an experienced ultrasound examiner using standardized examination techniques. The research revealed that 18.1% of dermoid cysts were misdiagnosed, 6% of which were misdiagnosed as endometrioma. The rate of misdiagnosis may be even higher among less experienced radiologists.

Due to the similarities in ultrasound appearance between ovarian endometriomas and dermoid cysts in certain cases, and the subjective and observer-dependent nature of ultrasound image interpretation, accurate differential diagnosis of these two lesions poses challenges, particularly for junior radiologists. Recently, artificial intelligence (AI) technologies have offered advanced computational tools to complement the expertise of radiologists and have shown promising results in enhancing diagnostic capabilities for various diseases, including ovarian diseases (15–17). In this study, a diagnostic classification model based on ultrasound radiomics was developed to intelligently distinguish and diagnose the two diseases, and its diagnostic efficacy was compared to that of both senior and junior radiologists. To the best of our knowledge, this specific subject has been rarely investigated until now.

## 2 Materials and methods

### 2.1 Ethical approval

In this retrospective study, the use of previously obtained ultrasound images was approved by the ethical committees of the South China Hospital of Shenzhen University (approval number: HNLS20230112101-A). The requirement for patient informed consent was waived due to the retrospective nature of the study.

### 2.2 Study design and participants

This retrospective case–control study was conducted at the South China Hospital of Shenzhen University from June 2021 to October 2023. All participants underwent transvaginal or transrectal sonography scan at our hospital and were diagnosed as patients with ovarian endometrioma or ovarian dermoid cyst. Eventually, a total of 407 participants with 407 lesions were included in the study. Most of the masses were confirmed through pathological examination, while the remainder were determined based on expert judgment with imaging follow-up (lasting more than 6 months). The patients were randomly divided into a training cohort (*n* = 326) and a test cohort (*n* = 81), with an 8:2 ratio. Medical history of patients and clinical information such as age, maximum diameter of the lesion, and presenting symptoms were extracted from medical records. The symptoms included dysmenorrhea, chronic pelvic pain, dyspareunia, abdominal pain, and abdominal fullness.

The inclusion criteria for this study were as follows: (1) Patients diagnosed with either ovarian endometrioma or dermoid cyst, confirmed through either pathological examination or expert judgment with imaging follow-up (more than 6 months); (2) Availability of ultrasound images; (3) If the patients had multiple ovarian lesions, only the largest lesion was included. The exclusion criteria were as follows: (1) Low-quality ultrasound images that were unsuitable for further analysis; (2) Patients with inadequate clinical information.

### 2.3 Image acquisition

Ultrasound images, in JPG format, were extracted from datasets. The ultrasound examinations were conducted in a transvaginal manner for non-virgins and in a transrectal manner for women with an intact hymen, utilizing various equipment such as the Mindray DC-80 and GE Voluson E10. Image quality control measures were implemented to exclude images of low quality. All images were obtained by certified radiologists, each having more than 3 years of independent diagnostic experience in pelvic ultrasonography. On average, four images per patient with ovarian lesions were collected, and the image displaying the maximum lesion diameter was ultimately selected for analysis.

### 2.4 Image segmentation and feature extraction

We used ITK-SNAP software (Version 3.8.0, USA) to manually segment regions of interest. Two experienced radiologists independently performed the segmentation for all lesions. Calculation of intraclass correlation coefficient (ICC) ≥ 0.75 was considered indicative of robustness. Feature extraction was conducted using an in-house program implemented in Pyradiomics.^1^ The handcrafted features can be categorized into three groups: (1) geometry, (2) intensity, and (3) texture. Geometry features describe the shape characteristics of the lesions. Intensity features depict the first-order statistical distribution of the voxel intensities within the lesions. Texture features describe the patterns, or the second- and high-order spatial distributions of the intensities. Here the texture features are extracted using several different methods, including the gray-level co-occurrence matrix (GLCM), gray-level run length matrix (GLRLM), gray level size zone matrix (GLSZM) and neighborhood gray-tone difference matrix (NGTDM) methods.

### 2.5 Feature selection and model construction

We conducted Mann-Whitney *U*-test statistical test and feature screening for all radiomic features. Only radiomic features with the *P* value <0.05 were kept. For features with high repeatability, Spearman correlation analysis was performed and the correlation coefficients between features were calculated to evaluate their multi-collinearity. If the correlation coefficient between any two features was greater than 0.9, only one of the features was retained. To retain the ability to accurately depict features to the greatest extent, we used a greedy recursive deletion strategy for feature filtering, that is, the feature with the greatest redundancy in the current set is deleted each time. Additionally, we utilized the least absolute shrinkage and selection operator (LASSO) regression model to reduce the number of features for signature construction. Depending on the regulation weight λ, LASSO shrinks all regression coefficients toward zero and sets the coefficients of many irrelevant features exactly to zero. In order to determine the optimal λ, 10-fold cross-validation with minimum criteria was employed, where the final value of λ resulted in the minimum cross-validation error. The retained features with non-zero coefficients were used for regression model fitting and combined into a radiomics signature. Following this, a radiomics score for each patient was obtained by a linear combination of retained features weighed by their model coefficients. The Python scikit-learn package was employed for LASSO regression modeling.

After LASSO feature screening, the model construction and evaluation were carried out using the scikit-learn package in Python (version 3.70). We input the final features into the machine learning models such as Logistic Regression (LR), Support Vector Machine (SVM), k-nearest neighbor (KNN), LightGBM, Multi-Layer Perception (MLP) and so on to construct the models. To determine the optimal model hyper parameters for model fitting and obtain the final rad signature, the 5-fold cross-verification were performed. The diagnostic efficacy of the radiomic model was assessed in the test cohort, and receiver operating characteristic (ROC) curves were plotted for visual evaluation of the models’ diagnostic performance. Additionally, diagnostic indices, including area under curve (AUC), specificity, sensitivity, accuracy, positive predictive value (PPV), negative predictive value (NPV), and precision were also calculated.

A radiomic nomogram was developed by combining the radiomic signature with the clinical signature. The diagnostic performance of the radiomic nomogram was assessed in the test cohort using ROC curves. Calibration curves were plotted to evaluate the calibration efficiency of the nomogram, and Hosmer-Lemeshow analytical fit was employed to assess the calibration ability of nomogram. In addition, decision curve analysis (DCA) was used to evaluate the clinical utility of the predictive models.

### 2.6 Radiologist evaluation

Four radiologists were divided into two groups based on their years of experience in gynecological ultrasonography: senior radiologists (WC and HT with over 15 years of experience) and junior radiologists (BW and JZ with less than 5 years of experience). Each radiologist was tasked with independently interpreting the test cohort. After a period of 2 months, the junior radiologists were instructed to reevaluate each lesion with the assistance of the radiomic model.

### 2.7 Statistical analysis

The clinical features, including age, maximum diameter of the lesion, and presenting symptom, were analyzed using *t*-test, Mann–Whitney *U*-test, or Chi-square test to compare the clinical characteristics of the patients. A two-sided *P*-value <0.05 was used to determine statistical significance. Python (version 3.70) was employed to perform the ICCs, Spearman rank correlation test, *Z* score normalization, and LASSO regression analysis. Additionally, the DeLong testing method was utilized to compare the AUCs of clinical, radiomic, and nomogram models (18).

## 3 Results

### 3.1 Baseline characteristics of participants

**FIGURE 1:**
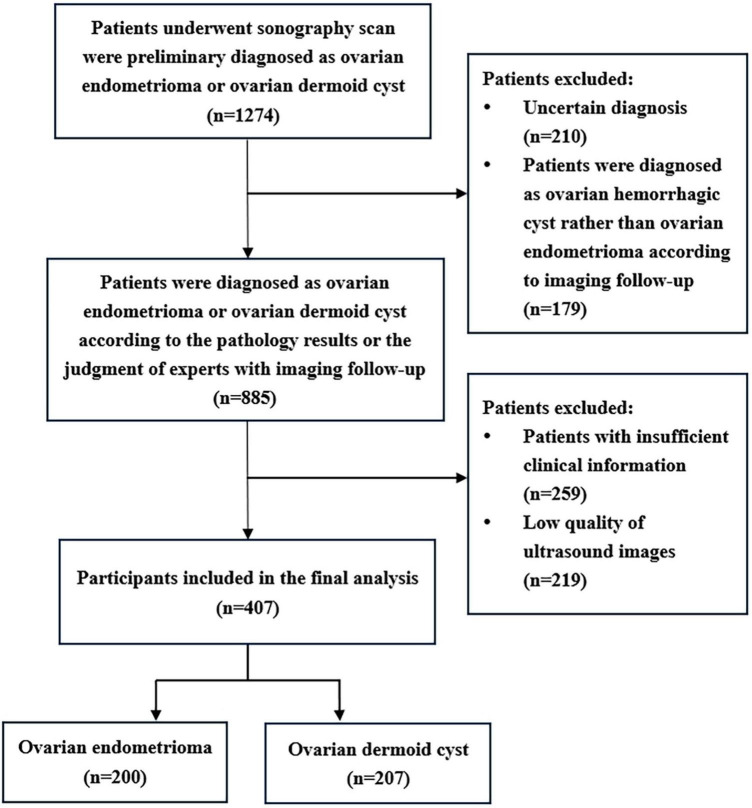
Flowchart of the study subjects screening based on inclusion and exclusion criteria.

A total of 407 participants with 407 lesions were analyzed and categorized into the ovarian endometriomas group (*n* = 200) and the dermoid cyst group (*n* = 207) based on pathology results or judgment of experts with imaging follow-up. Figure 1 illustrates the flowchart outlining the screening process of study subjects according to the inclusion and exclusion criteria. The mean age of the ovarian endometriomas group and the dermoid cyst group was 32.90 ± 6.82 years and 31.86 ± 8.15 years, respectively, (*P* = 0.098). The maximum diameter of the lesion in the ovarian endometriomas group and the dermoid cyst group was 40.22 ± 12.53 mm and 38.88 ± 11.27 mm, respectively, (*P* = 0.494). No significant differences were observed in terms of age and maximum diameter of the lesion between the two groups (*P* > 0.05). In the ovarian endometriomas group, 53.50% of participants were asymptomatic, whereas 46.50% had clinical symptoms. In the dermoid cyst group, 84.06% of participants were asymptomatic, while 15.94% had clinical symptoms. There was a significant difference in the proportion of symptomatic participants between the two groups (*P* < 0.05). Table 1 presents the baseline clinical characteristics of participants in both groups.

**TABLE 1:** Baseline clinical characteristics of participants between ovarian endometriomas group and dermoid cyst group.

Clinical features	All (*n* = 407)	Ovarian endometriomas group (*n* = 200)	Dermoid cyst group (*n* = 207)	*P* value
Age (years)	32.37 ± 7.53	32.90 ± 6.82	31.86 ± 8.15	0.098
Diameter (mm)	39.54 ± 11.89	40.22 ± 12.53	38.88 ± 11.27	0.494
Symptom				<0.001
0	281 (69.04%)	107 (53.50%)	174 (84.06%)	
1	126 (30.96%)	93 (46.50%)	33 (15.94%)	

Symptom 0 means the participants were asymptomatic. Symptom 1 means the participants had clinical symptoms.

### 3.2 Feature extraction and selection

**FIGURE 2:**
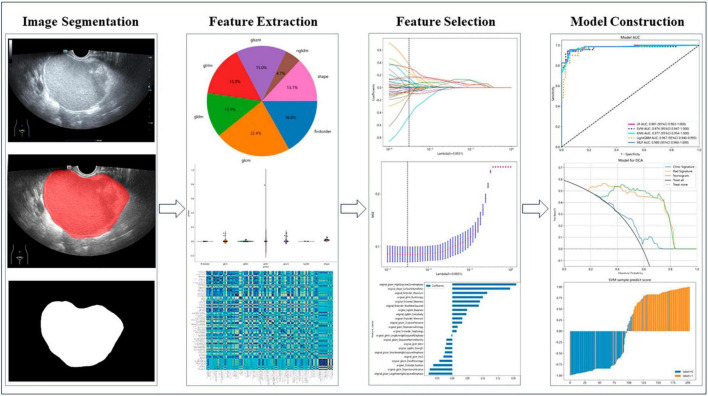
Workflow of ultrasound-based radiomic analysis.

**FIGURE 3:**
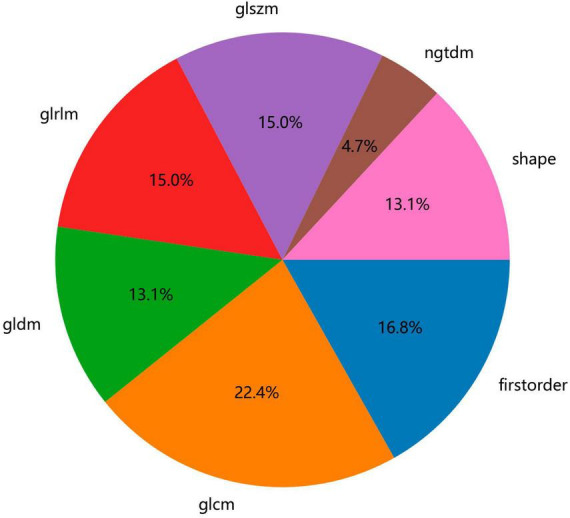
Number and ratio of handcrafted features.

**FIGURE 4:**
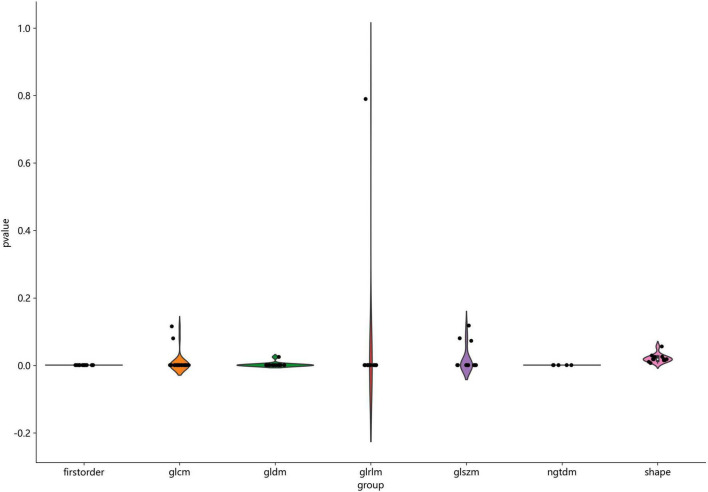
Statistics of radiomic features.

**FIGURE 5:**
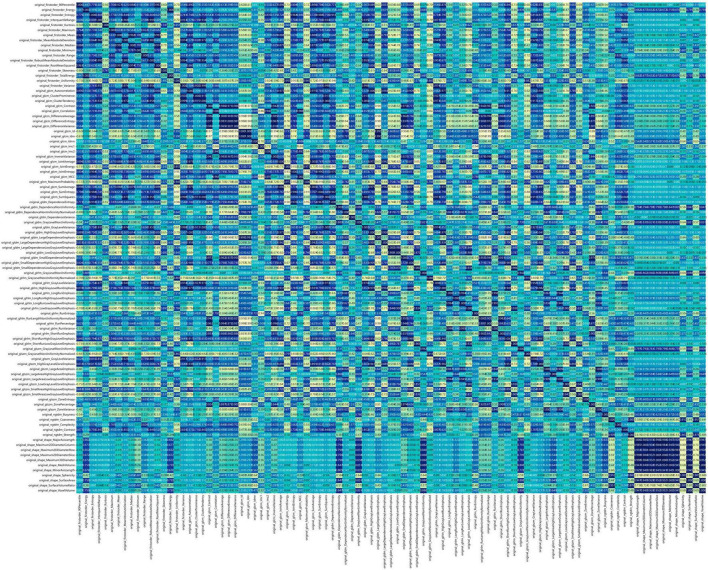
Spearman correlation coefficients between each feature.

Figure 2 shows the workflow of ultrasound-based radiomic model construction in this study. A total of 7 categories, 107 handcrafted features are extracted, including 18 first order features, 14 shape features, and 75 texture features. Details of the handcrafted features can be found in Figures 3–5. Figure 3 illustrates the count and proportion of handcrafted features, Figure 4 presents the statistics of radiomic features, and Figure 5 displays the Spearman correlation coefficients between each feature.

**FIGURE 6:**
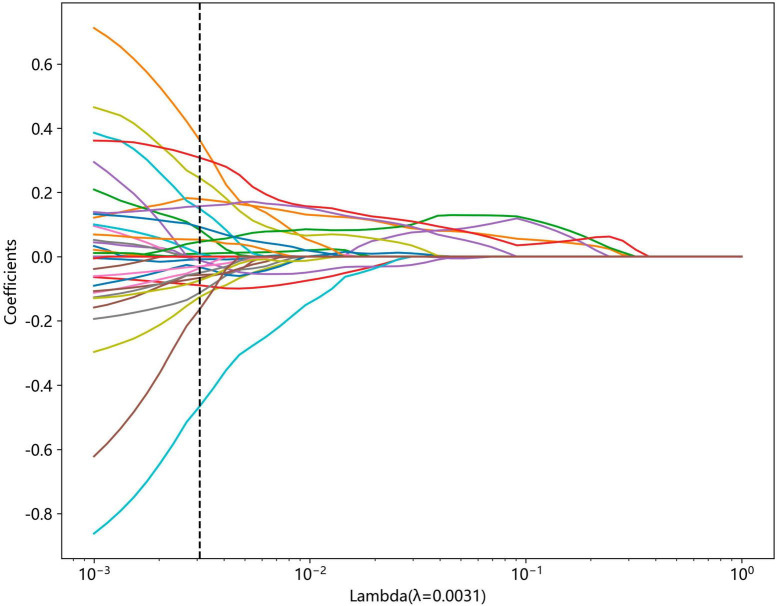
Coefficients of 10-fold cross-validation based on LASSO algorithm.

**FIGURE 7:**
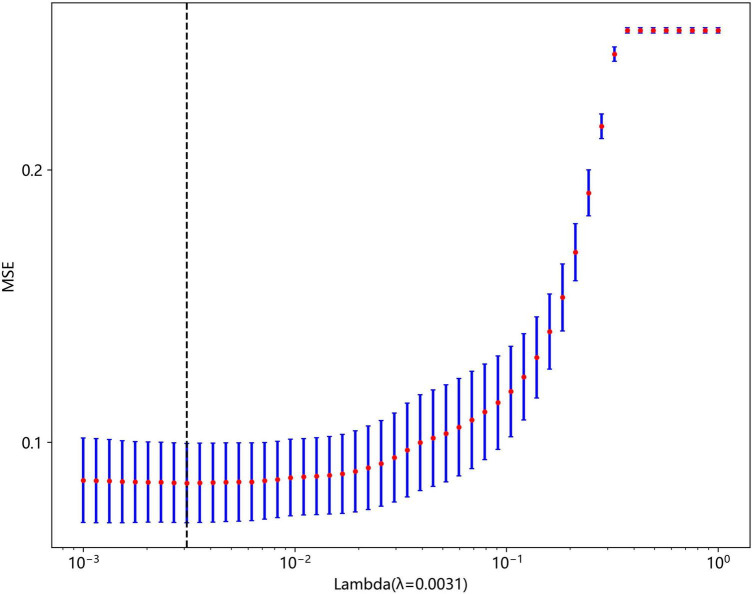
MSE of 10-fold cross-validation based on LASSO algorithm.

**FIGURE 8:**
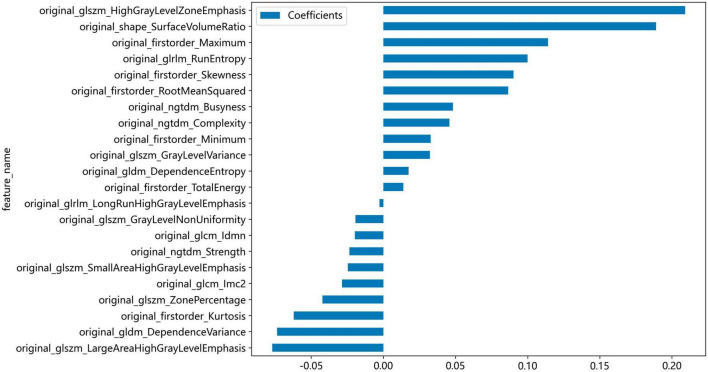
Histogram depicting the values of coefficients in the final selected non-zero features.

Non-zero coefficients were selected to establish the Rad-score through LASSO logistic regression model. A total of 22 features with non-zero coefficients were selected for the establishment of the Rad score using a LASSO logistic regression model. The coefficients and mean standard error (MSE) of 10-fold cross-validation are presented in Figures 6, 7, respectively. Figure 8 displays the histogram depicting the values of coefficients in the final selected non-zero features. All radiomic features were extracted, and prediction models were constructed based on the selected features.

### 3.3 Development and performance of radiomic models

**FIGURE 9:**
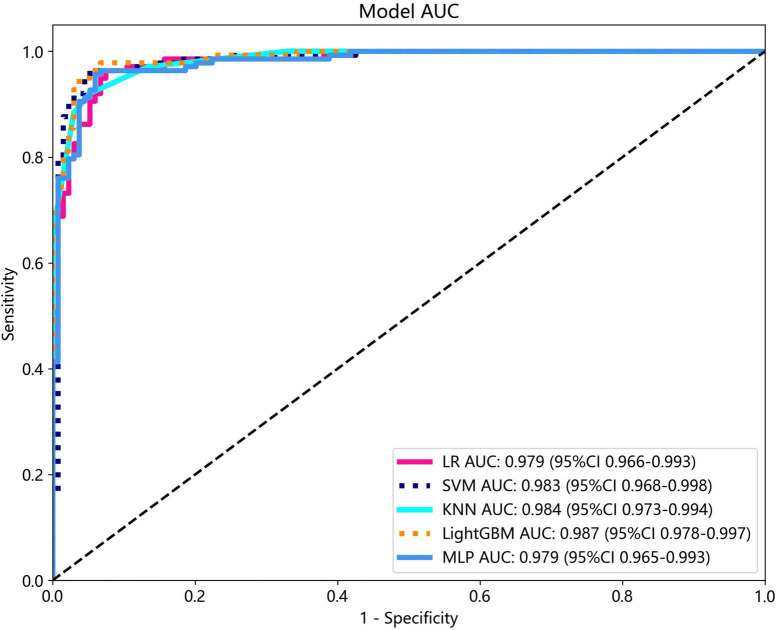
The ROC curves and AUC of different models in the training cohort.

**FIGURE 10:**
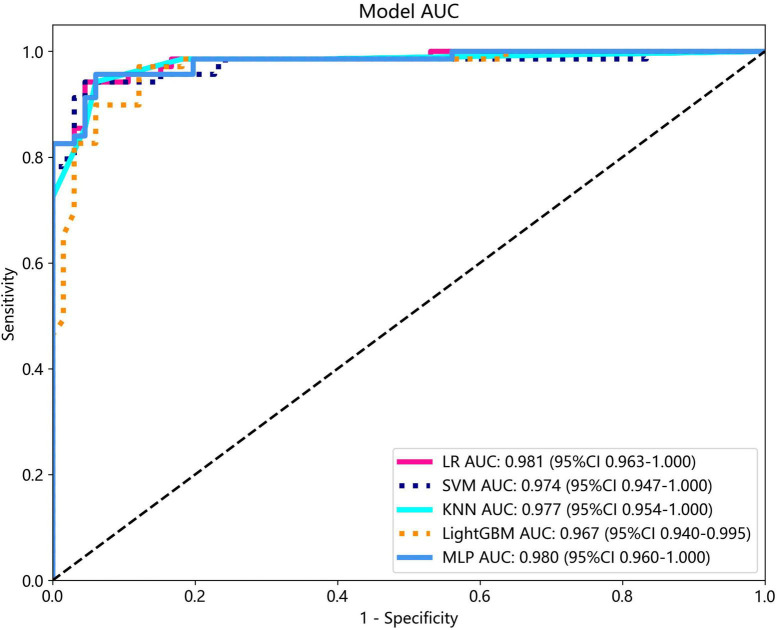
The ROC curves and AUC of different models in the test cohort.

Several models were constructed and compared to identify the most optimal performing model. The optimal model was obtained by utilizing rad features compared with LR, SVM, KNN, LightGBM, and MLP classifiers. LightGBM and LR achieved the highest AUC value of 0.987 (95% CI: 0.978−0.997) and 0.981 (95% CI: 0.963−1.000) in the training cohort and test cohort, respectively, for discriminating between ultrasound images of ovarian endometriomas and dermoid cysts. Diagnostic indices, including AUC, sensitivity, specificity, accuracy, PPV, NPV, and precision, for various models in the training and test cohorts are presented in Table 2. The ROC curves and AUC of different models in the training and test cohorts are shown in Figures 9, 10, respectively.

**TABLE 2:** Diagnostic performance of different models for discriminating between ultrasound images of ovarian endometriomas and dermoid cysts.

Cohort	Model	AUC (95% CI)	Accuracy (%)	Sensitivity (%)	Specificity (%)	PPV (%)	NPV (%)	Precision (%)
Train	LR	0.979 (0.966−0.993)	94.5	92.5	96.4	96.1	93.0	96.1
Train	SVM	0.983 (0.968−0.998)	95.6	94.8	96.4	96.2	95.0	96.2
Train	KNN	0.984 (0.973−0.994)	93.4	94.8	92.0	92.0	94.8	92.0
Train	LightGBM	0.987 (0.978−0.997)	95.6	96.3	94.9	94.9	96.3	94.9
Train	MLP	0.979 (0.965−0.993)	94.9	94.0	95.7	95.5	94.3	95.5
Test	LR	0.981 (0.963−1.000)	94.8	95.5	94.2	94.0	95.6	94.0
Test	SVM	0.974 (0.947−1.000)	94.8	95.5	94.2	94.0	95.6	94.0
Test	KNN	0.977 (0.954−1.000)	94.1	93.9	95.6	93.9	94.2	93.9
Test	LightGBM	0.967 (0.940−0.995)	92.6	87.9	97.1	96.7	89.3	96.7
Test	MLP	0.980 (0.960−1.000)	94.8	93.9	95.7	95.4	94.3	95.4

### 3.4 Performance comparison of clinical, radiomic, and nomogram models

**FIGURE 11:**
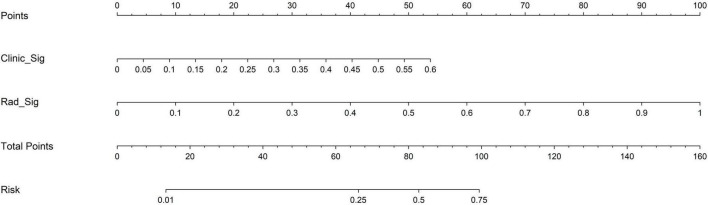
The nomogram with a total score reflecting the probability of ovarian dermoid cyst.

**FIGURE 12:**
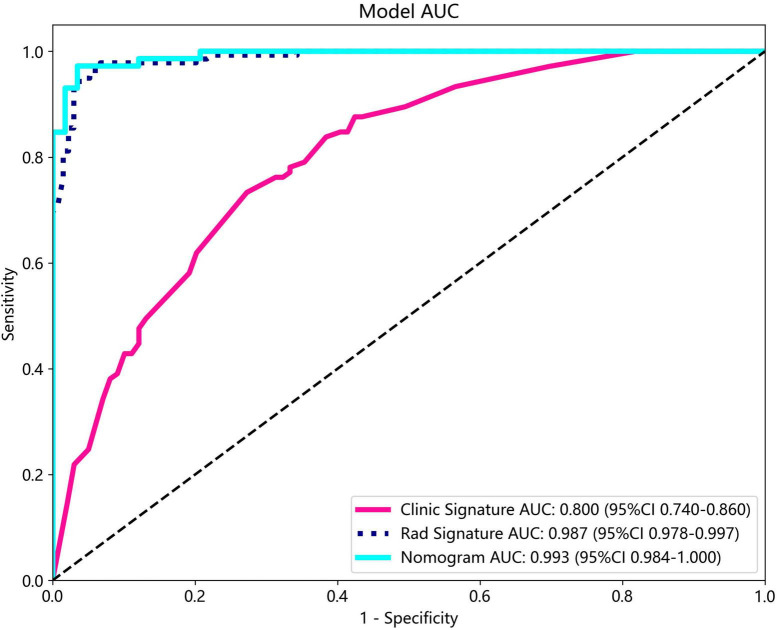
The ROC curves and AUC of the clinical, radiomic, and nomogram models in the training cohort.

**FIGURE 13:**
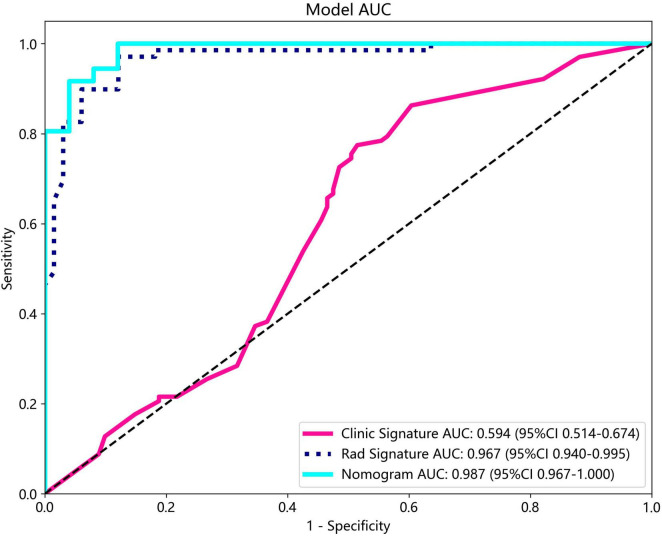
The ROC curves and AUC of the clinical, radiomic, and nomogram models in the test cohort.

The nomogram, using the LightGBM algorithm, incorporating both clinical features and radiomic features, demonstrated the highest level of performance (AUC: 0.987, 95% CI: 0.967−1.000, accuracy: 95.1%, sensitivity: 88.0%, specificity: 100.0%, PPV: 100.0%, NPV: 88.0%, precision: 93.6%). Table 3 presents the diagnostic indices, including AUC, sensitivity, specificity, accuracy, PPV, NPV, and precision of clinical, radiomic, and nomogram models in both the training and test cohorts. Figure 11 depicts the nomogram for clinical use, with a total score reflecting the probability of ovarian dermoid cyst. Figures 12, 13 illustrate the ROC curves and AUC values of the clinical, radiomic, and nomogram models in the training cohort and test cohort, respectively.

**TABLE 3:** Diagnostic performance of clinical, radiomic, and nomogram models.

Cohort	Model	AUC (95% CI)	Accuracy (%)	Sensitivity (%)	Specificity (%)	PPV (%)	NPV (%)	Precision (%)
Train	Clinical	0.800 (0.740−0.860)	73.0	72.7	73.3	72.0	74.0	72.0
Train	Radiomic	0.987 (0.978−0.997)	95.6	96.3	94.9	94.9	96.3	94.9
Train	Nomogram	0.993 (0.984−1.000)	96.9	96.6	97.2	96.6	97.2	96.6
Test	Clinical	0.594 (0.514−0.674)	63.1	48.5	79.8	68.1	60.3	68.1
Test	Radiomic	0.967 (0.940−0.995)	92.6	87.9	97.1	96.7	89.3	96.7
Test	Nomogram	0.987 (0.967−1.000)	95.1	88.0	100.0	100.0	88.0	93.6

The DeLong test revealed that there was no statistical difference in diagnostic performance between the radiomic model and nomogram model in the training cohort (*P* = 0.093) and in the test cohort (*P* = 0.131). Furthermore, both models demonstrated good performance in distinguishing ultrasound images of ovarian endometriomas and dermoid cysts.

**FIGURE 14:**
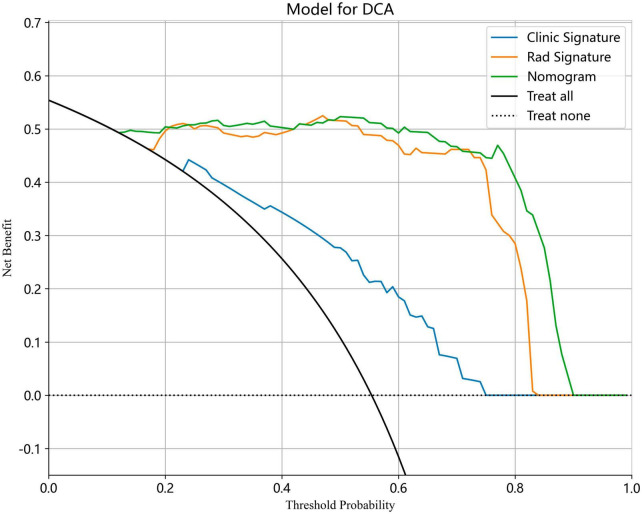
The DCA curves for clinical, radiomic, and nomogram models in training cohort.

**FIGURE 15:**
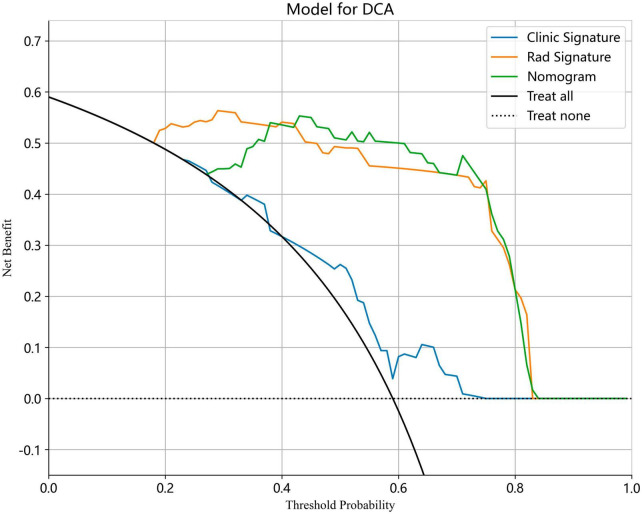
The DCA curves for clinical, radiomic, and nomogram models in test cohort.

We also evaluated the models through decision curve analysis (DCA). The result demonstrated that, when compared to scenarios without any prediction model, both the nomogram and radiomic models significantly enhanced the intervention outcomes for the participants. The prediction probability was 0.15−0.85 for the nomogram and radiomic models and 0.20−0.75 for the clinical model. These findings indicate that the use of radiomic and nomogram models in predicting ovarian endometriomas and dermoid cysts offers superior clinical benefits. Figures 14, 15 depict the DCA curves for clinical, radiomic, and nomogram models in training cohort and test cohort, respectively.

### 3.5 Performance comparison with radiologists

For senior radiologists, the average AUC, sensitivity, and specificity were 0.965 (95% CI: 0.948−0.981), 97.6%, and 95.5%, respectively. These values were comparable to those obtained by the radiomic models. In contrast, junior radiologists exhibited lower diagnostic performance, with an average AUC, sensitivity, and specificity of 0.907 (95% CI: 0.884−0.926), 90.8%, and 90.5%, respectively, when compared to the radiomic models. In the test cohort, junior radiologists with AI assistance displayed a significant improvement in diagnostic performance, achieving an average AUC, sensitivity, and specificity of 0.971 (95% CI: 0.953−0.989), 97.1%, and 97.0%, respectively. Supplementary Figure 1 illustrates the ROC curves and AUC values for senior radiologists, junior radiologists, and junior radiologists with AI assistance.

## 4 Discussion

Accurate differential diagnosis of ovarian endometrioma and dermoid cyst by sonography is crucial due to the distinct pathology, clinical presentations, and treatments associated with each condition. Endometrioma is characterized by the presence of endometrium-like epithelium and/or stroma, which undergoes cyclic bleeding (19). Women with endometrioma may experience different forms of pain (including dysmenorrhea, chronic pelvic pain, and dyspareunia) or be asymptomatic. Management options for endometriomas encompass expectant management, medical treatment, and surgical intervention (20). Surgical treatments involve techniques like such as cystectomy, electrocoagulation, ablation, ultrasound-guided aspiration and sclerotherapy, or combination of these methods (21). In contrast, dermoid cysts are composed of tissues derived from completely differentiated cells from ectodermal, mesodermal, or endodermal. Ectoderm, such as hair and other skin derivatives, are often present in dermoid cysts. They may also contain endoderm tissues (including thyroid or gastrointestinal tissue) or mesoderm tissues (including bone, fat, cartilage, or even neural tissue) (11). While most patients with dermoid cysts are asymptomatic, a small percentage may experience abdominal pain and abdominal fullness due to the presence of the mass (22). Surgical removal is an effective treatment for dermoid cysts with a diameter of around 5 cm in premenopausal women and a smaller size in the postmenopausal woman due to the heightened risk of malignant transformation (23).

The characteristic ultrasound feature of endometrioma is “unilocular cyst with ground glass echogenicity of the cyst fluid” (Supplementary Figure 2) while the typical ultrasound features of dermoid cysts are the presence of “dots and/or lines” and the “echogenic white ball” (Supplementary Figure 3). However, these diseases do not show typical ultrasound features. In some cases, the ultrasound appearance of ovarian endometrioma and dermoid cyst may exhibit overlapping features. The variability in the ultrasound appearance of endometriomas can be attributed to different stages of blood degradation (24) and occasionally, it can mimic the characteristics of a dermoid cyst. For instance, atypical ultrasound features of endometrioma include the presence of a “fluid-fluid level” and an avascular internal nodule or papillary projection, which are more frequently observed in postmenopausal women (25). During our clinical practice, we have witnessed confusion between the “fluid-fluid level” feature of endometrioma and the “fat-fluid level” feature of the dermoid cyst. Additionally, the avascular internal nodule or papillary projection of endometrioma may be misdiagnosed as the “echogenic white ball” feature of the dermoid cyst. The interpretation of ultrasound images heavily relies on the expertise of the radiologist. However, there is a relative shortage of experienced radiologists, and the human eye may struggle to differentiate subtle differences in the ultrasound features of these two conditions in certain cases. Therefore, it is necessary to use artificial intelligence technology to help reduce variability and ensure a more standardized approach across different observers.

To address this issue, we developed a machine learning model based on ultrasound radiomics to assist in the differential diagnosis of ovarian endometrioma and dermoid cyst in this study. The radiomics models showed satisfactory results in intelligently distinguishing between the two diseases. LightGBM and SVM achieved the highest AUC values of 0.987 (95% CI: 0.978−0.997) and 0.981 (95% CI: 0.963−1.000) in the training cohort and test cohort, respectively. The results demonstrated that this diagnostic model exhibited superior performance compared to junior radiologists and comparable performance to senior radiologists in both the training cohort and test cohort. More importantly, the diagnostic indexes for junior radiologists, such as AUC, sensitivity, and specificity, exhibited remarkable improvements when assisted by the AI model. The results showed increased values for AUC (0.907 vs. 0.971), sensitivity (90.8% vs. 97.1%), and specificity (90.5% vs. 97.0%). These findings illustrated that the implementation of this model can enhance the diagnostic performance of junior radiologists. The radiomic nomogram was established in combination with radiomic signature and clinical signatures. Due to the retrospective nature of the study, collecting clinical information proved challenging, resulting in only the extraction of patient age, maximum lesion diameter, and presenting symptoms in this study. The results revealed that only the proportion of symptomatic participants demonstrated significant difference between the two groups (*P* < 0.05). Due to the lack of informative clinical features, the nomogram did not yield a substantial improvement in diagnostic performance compared to the radiomics model. The DeLong test indicated no statistical difference in diagnostic performance between the radiomic model and the nomogram model. In future studies, a wider range of clinical features will be collected to enhance the diagnostic performance of the nomogram. Moreover, the results of DCA demonstrated that the utilization of both radiomic and nomogram models can provide valuable clinical insights for guiding treatment decisions.

Radiomics involves the conversion of medical images into high-dimensional quantitative data, enabling the extraction of comprehensive sets of quantitative signatures that characterize microscopic tissue aspects (26). These data can subsequently be analyzed using either conventional biostatistics or AI methods (27). By utilizing sophisticated image processing techniques, all medical images are transformed into mineable high-throughput image features, which can further correlate with pathology diagnoses based on these processed feature signatures (28). A number of studies have been published regarding the AI assisted diagnosis for ovarian masses (29–31). However, these studies have predominantly focused on distinguishing between benign and malignant masses (15, 32). For example, Gao et al. (33) devised a deep convolutional neural network model which automated the detection of adnexal masses in ultrasound images to distinguish between malignant and benign masses. This model exhibited a higher accuracy rate compared to radiologists and matched the level of expert ultrasound image readers. Zhang et al. (34) and Wang et al. (35), respectively, developed radiomics and a deep learning algorithm to differentiate benign and malignant ovarian lesions on routine MRI. The results indicated that AI technologies could assess the nature of ovarian masses on MRI with a higher level of accuracy and specificity than radiologists. Additionally, several studies (36–39) have further used AI technologies to discriminate between borderline and malignant ovarian tumors observed on ultrasound or MR images. These models have shown promising diagnostic efficiency and provided complementary clinical diagnostic information. Nevertheless, there has been a notable scarcity of research centered on the differential diagnosis of benign ovarian lesions. Only Ştefan et al. (4) and Lupean et al. (40) reported the differential diagnosis of endometriomas and functional hemorrhagic cysts on ultrasound and MR images. As far as we know, this is the first study concentrating on the discrimination between ultrasound images of ovarian endometrioma and dermoid cyst using AI technology.

Notwithstanding the utility, machine learning methods are known to have some limitations (41) and this study has following limitations: (1) the sample size of our dataset is relatively small, giving rise to potential selection bias; (2) human error is unavoidable in the manual delineation of lesion boundaries, leading to potential omission of some characteristics; (3) the radiomics model was developed using retrospective data, and not all data are confirmed by pathology; (4) our models were developed solely using ultrasound data, and the inclusion of additional medical images or clinical signatures may enhance their performance; (5) this study was conducted at a single center. In the future, larger multicenter prospective trials incorporating a broader range of clinical features will be necessary to enhance the clinical evidence and evaluate the effectiveness of our diagnostic model in clinical practice.

## 5 Conclusions

We initially developed a diagnostic model based on ultrasound radiomics that exhibited a satisfactory predictive ability in distinguishing between ovarian endometrioma and ovarian dermoid cyst. The model demonstrated superior diagnostic performance compared to that of junior radiologists and comparable diagnostic performance to that of senior radiologists. Moreover, the utilization of this model has the potential to enhance the diagnostic performance of junior radiologists when it comes to ovarian lesion diagnosis, while also providing valuable clinical insights for guiding treatment decision.

## Data availability statement

The raw data supporting the conclusions of this article will be made available by the authors, without undue reservation.

## Ethics statement

The studies involving humans were approved by the study was conducted in accordance with the Declaration of Helsinki and approved by the ethical committees of the South China Hospital of Shenzhen University (approval number: HNLS20230112101-A). Patient consent was waived by the ethical committees due to the retrospective nature of the study. The studies were conducted in accordance with the local legislation and institutional requirements. Written informed consent for participation was not required from the participants or the participants’ legal guardians/next of kin in accordance with the national legislation and institutional requirements.

## Author contributions

LL: Methodology, Investigation, Formal Analysis, Conceptualization, Writing – original draft. WC: Writing – review & editing, Formal Analysis, Data curation, CZ: Writing – original draft, Validation. HT: Writing – review & editing. BW: Writing – review & editing. JZ: Writing – review & editing. GY: Writing – review & editing. YH: Writing – review & editing.

## Footnotes

1
http://pyradiomics.readthedocs.io

## Supplementary material

Supplementary Figure 1 The ROC curves and AUC for senior radiologists, junior radiologists, and junior radiologists with AI assistance.

Supplementary Figure 2 The typical ultrasound feature of endometrioma is “unilocular cyst with ground glass echogenicity of the cyst fluid.”

Supplementary Figure 3 The typical ultrasound features of dermoid cysts are “dots and/or lines” **(A)** and “echogenic white ball” **(B)**.
