# Supplementary figures and images for: Ultrasound radiomics-based artificial intelligence model to assist in the differential diagnosis of ovarian endometrioma and ovarian dermoid cyst
Source: Front Med (Lausanne). 2024 Mar 8;11:1362588. doi: 10.3389/fmed.2024.1362588 (PMC10957533; doi:10.3389/fmed.2024.1362588)

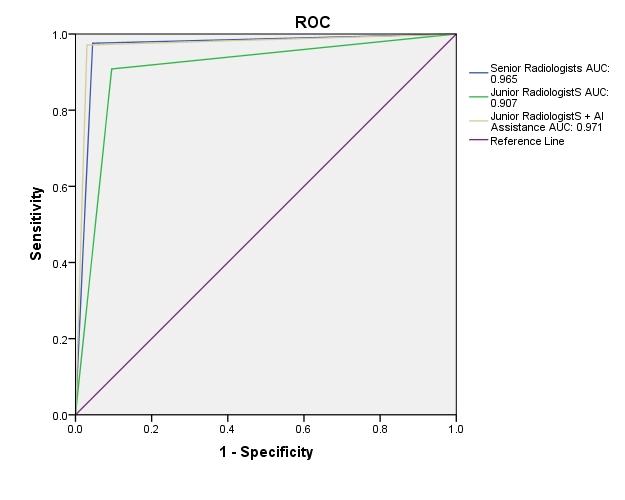

Supplement: Supplementary Figure 1 — The ROC curves and AUC for senior radiologists, junior radiologists, and junior radiologists with AI assistance. [file Image_1.JPEG]

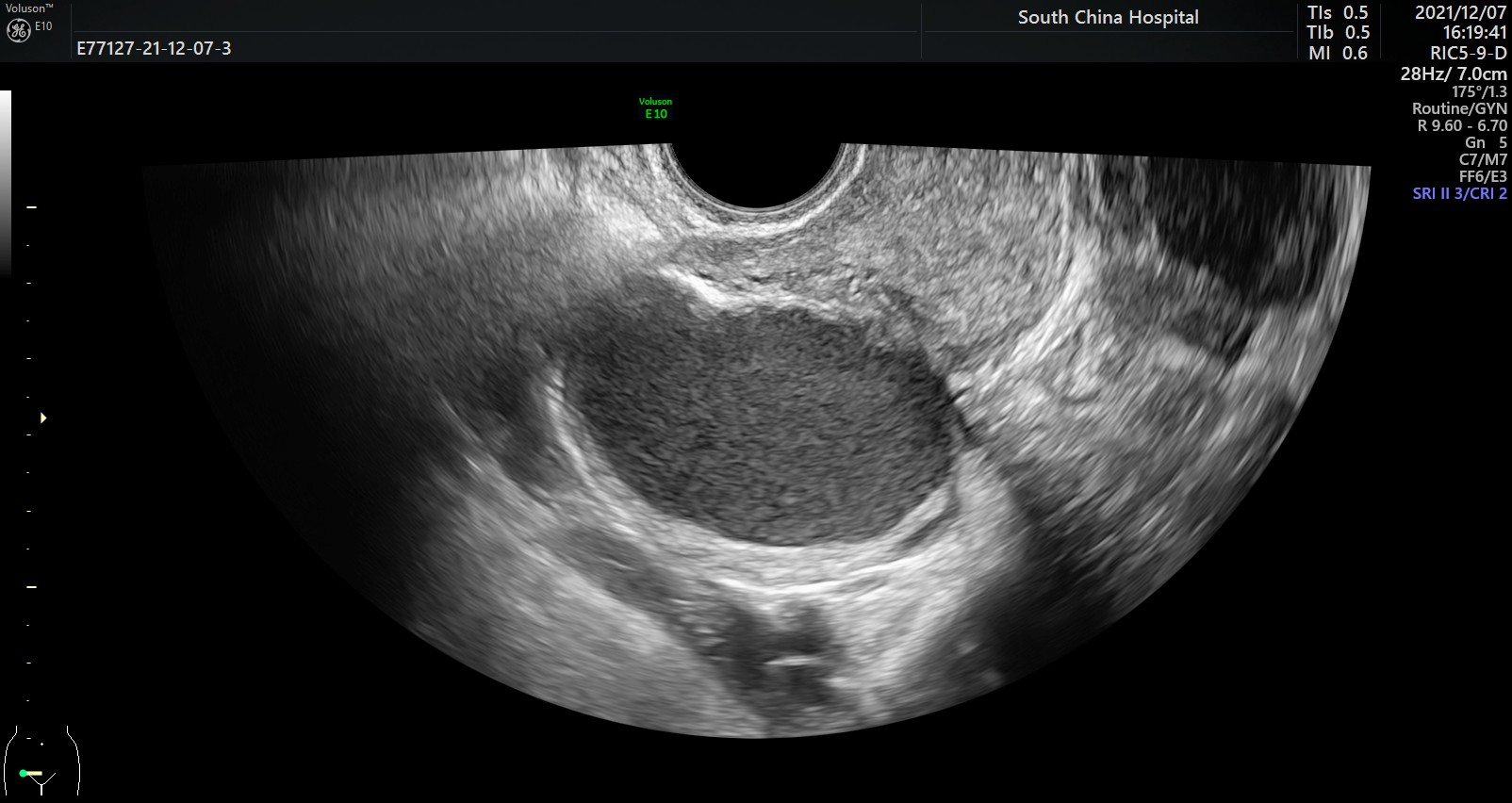

Supplement: Supplementary Figure 2 — The typical ultrasound feature of endometrioma is “unilocular cyst with ground glass echogenicity of the cyst fluid.” [file Image_2.JPEG]

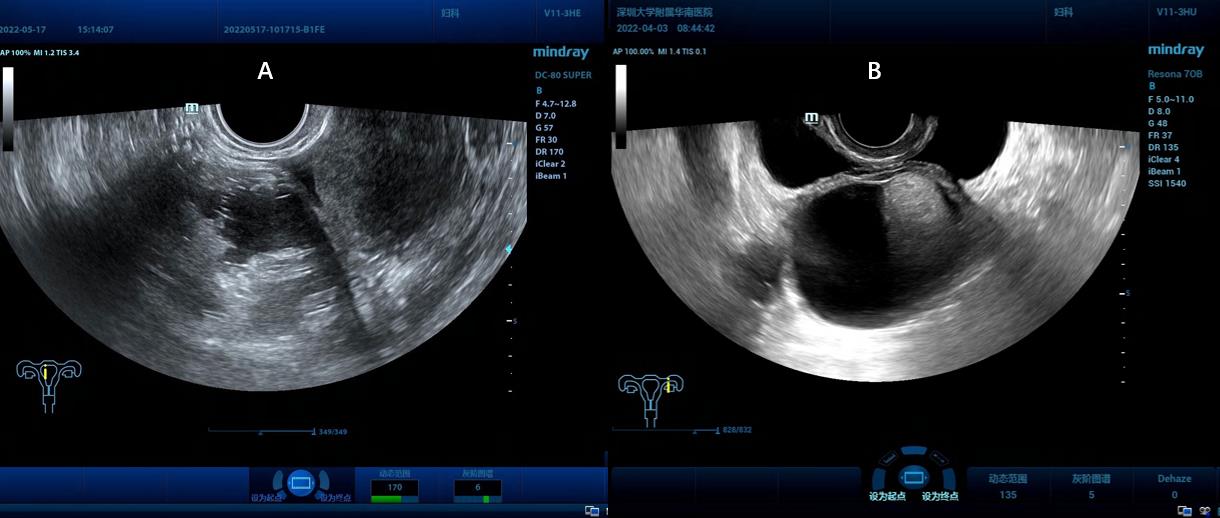

Supplement: Supplementary Figure 3 — The typical ultrasound features of dermoid cysts are “dots and/or lines” (A) and “echogenic white ball” (B). [file Image_3.JPEG]
